# The association of gabapentin initiation and neurocognitive changes in older adults with normal cognition

**DOI:** 10.3389/fphar.2022.910719

**Published:** 2022-11-25

**Authors:** GYeon Oh, Daniela C. Moga, David W. Fardo, Erin L. Abner

**Affiliations:** ^1^ Sanders-Brown Center on Aging, University of Kentucky, Lexington, KY, United States; ^2^ Department of Epidemiology and Environmental Health, University of Kentucky, Lexington, KY, United States; ^3^ Department of Pharmacy Practice and Science, University of Kentucky, Lexington, KY, United States; ^4^ Institute for Pharmaceutical Outcomes and Policy, University of Kentucky, Lexington, KY, United States; ^5^ Department of Biostatistics, University of Kentucky, Lexington, KY, United States

**Keywords:** gabapentin, older adults, NACC data, cognitive decline, functional status change, motor function change

## Abstract

**Background:** Gabapentin is increasingly prescribed to older adults, which raises concerns about its potential to cause neurocognitive changes. Therefore, we aimed to examine the association of gabapentin use with neurocognitive changes (i.e., cognitive decline, functional status decline, and motor function change) in older adults.

**Methods:** We conducted a retrospective cohort study using the National Alzheimer’s Coordinating Center Uniform Data Set (UDS; September 2005-March 2021 data freeze). From the eligible sample (≥age 65 years), we identified cognitively normal new-users of gabapentin and the visit they initiated gabapentin (i.e., index visit). Initiators were matched to randomly selected nonusers on year of UDS enrollment and visit number from enrollment to index. Cognitive decline was defined as any increase in the Clinical Dementia Rating global score (CDRGLOB) and as a 1-point increase in CDR sum of boxes (CDR-SB). Functional status decline was defined as a 3-point increase in the sum of the Functional Activities Questionnaire (FAQ) and as 0.3-point increase in mean FAQ. Decline in motor function was defined as new clinician reports of gait disorder, falls, and slowness. To mitigate confounding and selection bias, we used joint stabilized inverse probability of treatment weights and stabilized inverse probability of censoring weights. All analyses were conducted comparing index to index+1 and index+2 visits.

**Results:** From the eligible UDS participants (N = 23,059), we included 480 initiators (mean age [SD]: 78.7 [6.9]; male 34.4%); 4,320 nonusers (78.3 [7.0]; 34.4%). Gabapentin initiation was significantly associated with cognitive/functional status decline: worsening CDRGLOB at index+1 visit (odds ratio [95% confidence interval]: 1.55 [1.07, 2.25]); CDR-SB at index+1 visit (1.94 [1.22, 3.09]); and mean of FAQ at index+2 visit (1.78 [1.12, 2.83]). After excluding initiators with extant motor dysfunction (n = 21), we identified 459 initiators (78.7 [6.9]; 34.0%) and 4,131 nonusers (78.2 [6.9]; 34.7%); in this sample, gabapentin initiation was associated with increased falls at the index+2 visit (2.51 [1.19, 5.31]).

**Conclusion:** Gabapentin initiation was significantly associated with deleterious neurocognitive changes among older adults with initially normal cognition. Further studies are needed to examine the risk/benefit of prescribing gabapentin in older adults.

## 1 Introduction

Gabapentin was first approved by the United States (US) Food and Drug Administration (FDA) in 1993 to treat partial seizures and additionally approved for postherpetic neuralgia in 2004 (Pfizer, 2017). By 2018, gabapentin was the 6^th^ most prescribed medication in the US market (IQVIA Institute for human data science, 2019). Increasing evidence suggests potential for gabapentin misuse and related adverse events (e.g., respiratory depression, sedation, physical dependence, and depression) (Gomes et al., 2017; Slavova et al., 2018; Tomko et al., 2018; McAnally et al., 2020; Evoy et al., 2021). Therefore, FDA announced a safety concern of severe breathing difficulties when gabapentin is used concurrently with opioids and other CNS depressants (U.S. Food and Drug Administration, 2019). Further, gabapentin was added to the American Geriatrics Society Beers criteria in 2019 as a medication to avoid using with opioids due to higher risks of sedation, respiratory depression, and death (By the 2019 American Geriatrics Society Beers Criteria® Update Expert Panel, 2019).

Older adults have age-related decreases in liver and kidney function (ElDesoky, 2007) and have a high chance of polypharmacy (Charlesworth et al., 2015), thus they could be more vulnerable to adverse effects associated with gabapentin. Using Medical Expenditure Panel Survey data, Johansen et al. reported that gabapentin use increased from 2002 to 2015 in adults age 65 and older (Johansen, 2018). Most gabapentin prescribing is known to be off-label indications, such as neuropathic pain, migraines, substance use disorder, and treatment for psychiatric symptoms (Botts and Raskind, 1999; Frye et al., 2000; Pande et al., 2000; Mathew et al., 2001; Gentry et al., 2002; Moore et al., 2018). Especially in older adults, gabapentin is prescribed to treat behavioral and psychological symptoms of dementia (BPSD) (Kim et al., 2008). Several studies have reported that gabapentin has a deleterious effect on cognition (Leach et al., 1997; Meador et al., 1999; Shem et al., 2018). A prospective observational cohort study has reported that gabapentin initiators with spinal cord injury had a cognitive decrease using neuropsychological tests. However, this study had a small sample size and no control group (Shem et al., 2018). In a randomized crossover study, gabapentin use was associated with worse attention/vigilance, ability to voluntarily maintain wakefulness, and cognitive processing and motor speed in healthy adults (Meador et al., 1999). However, other randomized studies (length of follow-up: 2 weeks (minimum); 26 weeks (maximum)) have reported that gabapentin was not associated with cognitive decline or impairment in patients with partial seizures (Dodrill et al., 1999) nor in healthy adults (Martin et al., 1999; Salinsky et al., 2002).

The association of gabapentin use and neurocognitive change is not well understood, and given how frequently gabapentin is prescribed, it is important to fully examine the benefit and risk of gabapentin use in older adults. The aim of this study was to estimate the association of gabapentin initiation with changes in cognitive status, functional status, and motor changes up to 2 years later, in older adults with initially normal cognition.

## 2 Methods

### 2.1 Data set and participants

**FIGURE 1:**
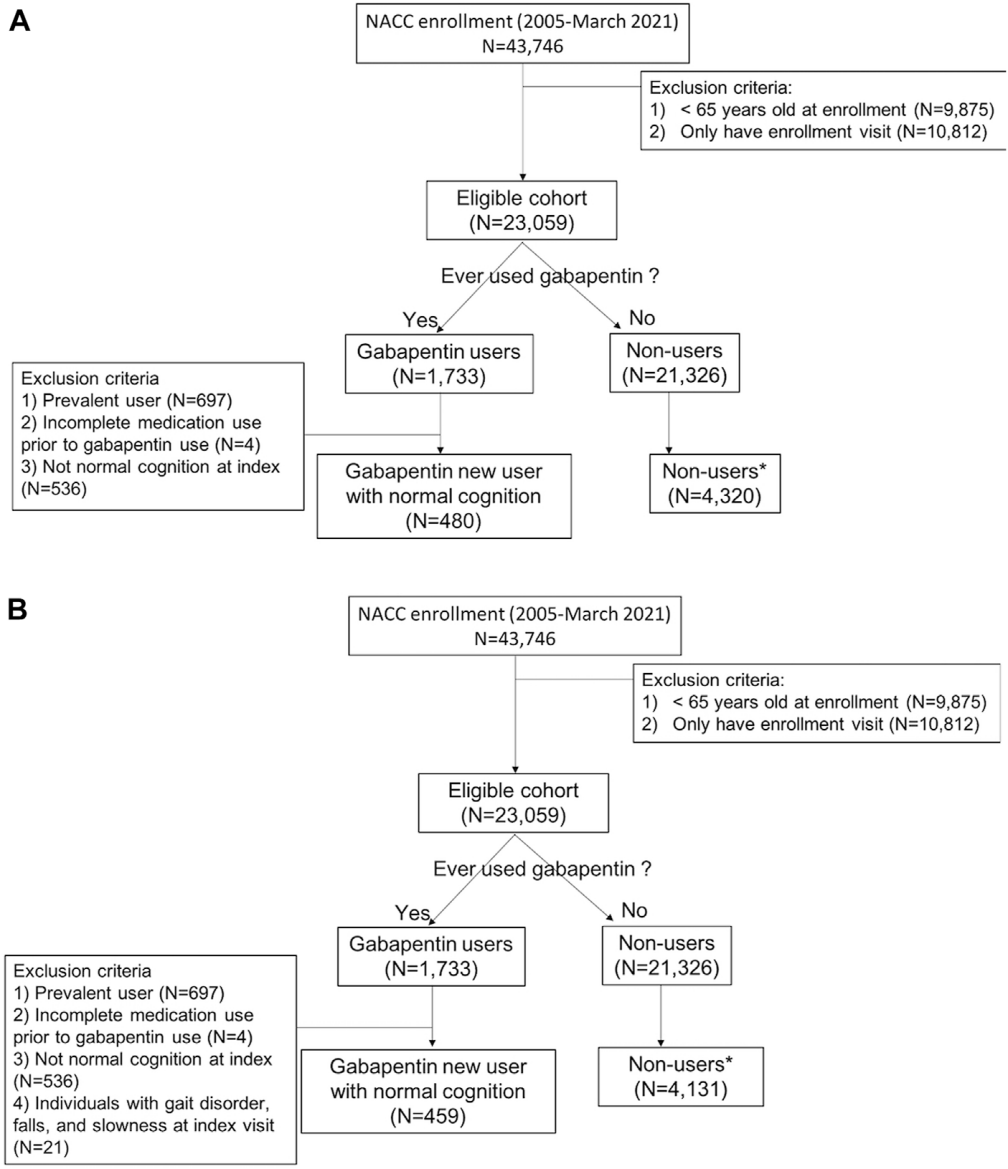
Flow diagram of inclusion and exclusion criteria **(A)** for measuring cognitive and functional status decline and **(B)** for measuring motor function change*1 to 9 randomly selected matched by year of first enrollment and visit number to gabapentin initiation from the enrollment.

The study data was drawn from the National Alzheimer’s Coordinating Center’s (NACC) Uniform Data Set (UDS) from 2005 to March 2021. NACC was first established in 1999 to aggregate and share data collected at National Institute on Aging-funded Alzheimer’s Disease Research Centers (ADRCs) and conduct research related to AD (Morris et al., 2006). Beginning in September 2005, a standard data collection protocol called the Uniform Data Set (UDS) was implemented at all ADRCs. Currently, there are more than 1100 published studies using NACC data which is collected across 26 states (The National Alzheimer’s Coordinating Center, 2022). Data submitted to NACC undergo a robust quality control process that assesses conflicting, missing, and impossible values, both within and across study visits, before they are shared with researchers. Approximately annually the participants’ information, such as demographics, medical history, cognitive and functional status, and behavioral symptoms, was collected by trained interviewers and clinicians; participants comprise a range of cognitive status, including normal cognition, mild cognitive impairment (MCI), and dementia. For the current study, participants 65 years and older at the time of enrollment in one of the 42 participating ADRCs were included (Figure 1).

### 2.2 Study design

**FIGURE 2:**
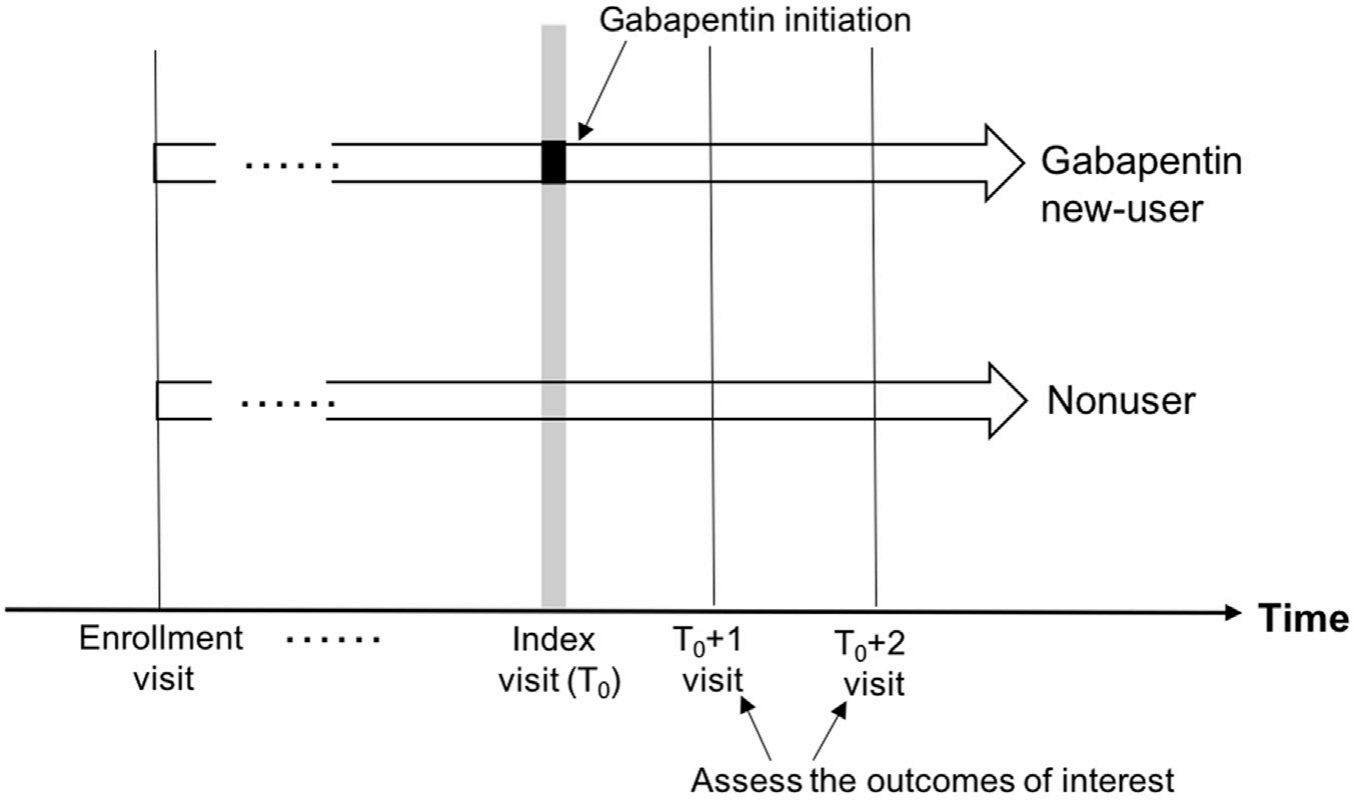
Depiction of study design comparing gabapentin initiators and nonusers.

From the eligible cohort, we selected all participants who reported use of gabapentin, and the index visit was defined as the first reported gabapentin use (Figure 2). We next excluded gabapentin users who: 1) reported gabapentin use at their first UDS visit (prevalent users); 2) had incomplete medication information prior to the index visit; and 3) had any syndromic cognitive diagnosis other than “normal” at the index visit. We excluded the prevalent users to minimize prevalent-user bias, and we implemented a new-user design (Ray, 2003). Once the gabapentin initiators were identified, non-users were randomly selected (1 to 9 ratio) with replacement (i.e., a non-user was allowed to be selected as a comparator multiple times, with each being assigned a different index visit). To minimize bias (e.g., secular trends, survival bias, and attrition bias) due to the long duration of study time (2005–2021), non-users were matched on the year of first enrollment and the number of visits from enrollment to gabapentin initiation to new-users. Non-users were subject to the same exclusion criteria as the gabapentin new-users. For the analyses of motor change, we selected a separate cohort, imposing the additional restriction of no reported motor dysfunction at the index visit [Figure 1, panels A (cognitive and functional outcomes) and B (motor outcomes)].

### 2.3 Gabapentin use determination

Medication use in the UDS is operationalized *via* an interview that asks participants to report all medications, including prescriptions and over-the-counter medications, they have used in the 2 weeks preceding their annual study visit. Gabapentin use was defined as any reported use of gabapentin. Data on indication and dose are not available in the NACC data.

### 2.4 Outcomes description

The outcomes of interest, including cognitive decline, functional status decline, and motor function change, were measured at the first (approximately 1 year later: index+1) and second (approximately 2 years later: index+2) follow-up visits after the index visit (Figure 2).

#### 2.4.1 Cognitive decline

Global cognition, measured by the Clinical Dementia Rating global score (CDRGLOB) and sum of boxes (CDR-SB), was assessed at each UDS visit. CDRGLOB is a ordinal rating with five levels (0: no dementia; 0.5: questionable dementia; 1: mild dementia; 2: moderate dementia; and 3: severe dementia) (Morris, 1993). CDR-SB is the sum of the six domain scores (memory, orientation, judgment and problem solving, community affairs, home and hobbies, and personal care; range from 0 to 18). With the goal of detecting clinically significant decline, we used two definitions to classify participants as showing cognitive decline: compared to the index visit (1) a higher score of CDRGLOB and (2) a 1-point increase of CDR-SB at follow-up. These definitions were based on a previous analysis of the NACC dataset that determined clinically important changes in cognitive status (Andrews et al., 2019).

#### 2.4.2 Functional status decline

Functional status was measured with the Functional Activities Questionnaire (FAQ). The participants or co-participants were asked whether the participant had any difficulty or needed help with ten instrumental activities of daily living (e.g., paying bills, assembling tax records, shopping alone for groceries, playing games, turning off the stove, preparing a balanced meal, keeping track of current events, understanding a TV program, remembering appointments, and driving). Each category was scored as 0: normal; 1: has difficulty, but does by self; 2: requires assistance; and 3: dependent. The functional status of each participant in this study was measured through total FAQ score, which includes the participants who did not have missing in any of the ten categories (72% of the total study sample), and the mean of FAQ score, which includes the participants who had a score in any of ten categories (Teng et al., 2010). The participants were categorized as showing functional status decline from the index to the first follow-up or second follow-up after the index visit if they had at least a 3-point increase in their sum of FAQ (Andrews et al., 2019) or 0.3 points increase in their mean of FAQ.

#### 2.4.3 Motor function change

Motor change was measured by clinician ratings of gait disorder, falls, and slowness.

### 2.5 Covariates

Confounders included in the propensity score model for inverse probability of treatment weighting were selected using directed acyclic graphs (DAG) (Textor et al., 2011). For measuring cognitive and functional status decline, confounders included demographics (age, sex, education, and race); body mass index (BMI); smoking history; comorbidities (depression, diabetes, and hypertension); medications (opioids, antipsychotics, and benzodiazepines); and *APOE* e4 allele status (Supplementary Figure S1). For measuring motor function change, confounders included demographics (age, sex, education, and race); body mass index; smoking history; comorbidities (depression, diabetes, Parkinson’s disease, and anxiety); and medications (opioids, antiseizures, and anxiolytic, sedative, and hypnotics) (Supplementary Figure S2).

The baseline characteristics (except for smoking history [at least 100 cigarettes over lifetime], diabetes, and hypertension) of gabapentin initiators and non-users were measured at the index visit. Some medical history variables (smoking history, diabetes, and hypertension) were only collected at the first UDS visit for each participant. Detailed descriptions for covariates included in this study are in the supplementary (Supplementary Table S1). Briefly, medical history was collected at annual visits *via* a structured interview with a study clinician, querying participants on the presence of diagnosed medical conditions.

### 2.6 Statistical analysis

To mitigate confounding and selection bias, we used joint stabilized inverse probability of treatment weights (SIPTW) and stabilized inverse probability of censoring weights (SIPCW) (Austin and Stuart, 2015). Using SIPTW, we created a balanced distribution of the measured confounders between the initiators and non-users. Since some participants had no follow-up visit after the index (i.e., censored), we generated SIPCW, and the SIPCW were multiplied by SIPTW to obtain joint weights. The weighted population is called the “pseudo-population”. In the pseudo-population, the conditional probability of gabapentin initiation is independent of the measured confounders, and the conditional probability of having a follow-up visit is independent of the confounders and gabapentin initiation (Austin and Stuart, 2015). We assessed the success of the weighting procedure by examining the distribution of the weights as well as the standardized mean differences of the measured confounders between initiators and non-users in unweighted and weighted samples (Supplementary Table S2). To obtain robust standard errors to account for the weighting, as well as to account for within-participant correlation for nonusers matched to more than one initiator, we used generalized estimating equations with an exchangeable working correlation structure to fit logistic regression models to the data.

## 3 Results

### 3.1 Cognitive decline

**FIGURE 3:**
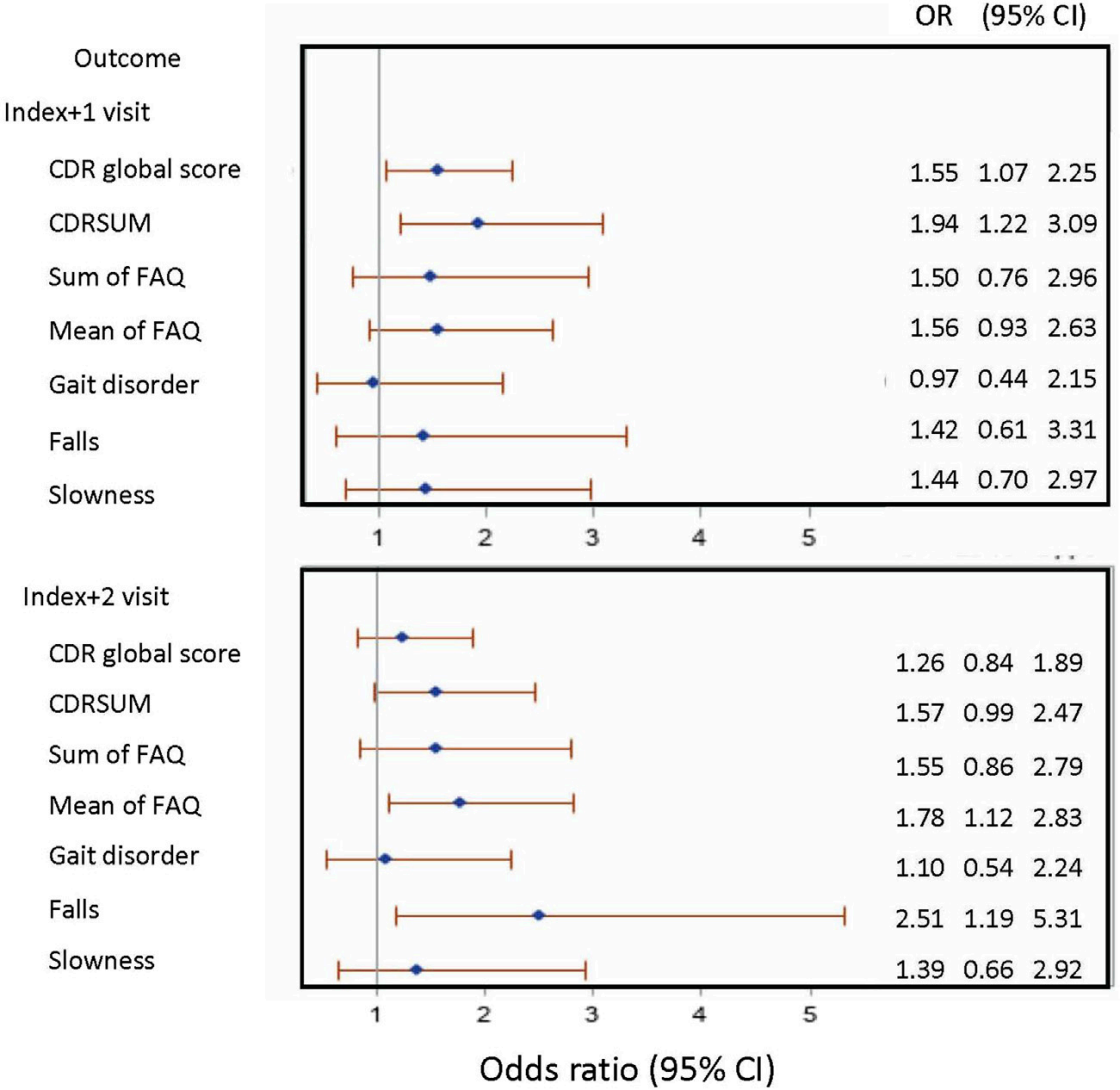
Forest plot presenting the odds ratio (95% confidence interval) of outcomes of interest in gabapentin new-users compared to non-users. Note: CDR: Clinical Dementia Rating; CDRSUM: Clinical Dementia Rating sum of boxes; FAQ: Functional Activities Questionnaire; OR: odds ratio; CI: confidence interval.

Among eligible ADRC participants with UDS data (N = 23,059), 480 gabapentin new-users (mean age [SD]: 78.7 [6.9]; male gender = 34.4%) were identified, and 4,320 nonusers (78.3 [7.0]; 34.4%) were randomly selected (Figure 1A). The mean (SD) number of annual visits between the first UDS visit and the index visit was 4.8 (2.8). Gabapentin initiators had less educational attainment (36.0% vs. 42.2% for graduate degree), had more comorbidities (diabetes: 22.1% vs. 11.9%; hypertension: 63.8% vs. 56.5%; depression: 12.9% vs. 7.9%), were taking more medications (opioids: 20.4% vs. 4.1%; benzodiazepines: 10.8% vs. 5.2%), and had higher BMI (≥30: 29.2% vs. 21.3%) compared to nonusers (Table 1). Among nonusers, 79.6% and 62.1% had the first and the second follow-up visit after index, respectively. Among gabapentin new-users who had the first (N [%]: 383 [79.8%]) and second (280 [58.3%]) follow-up visits after index, 58.5% and 50.0% reported gabapentin use at the first and second follow-up visits after index, respectively, After applying the joint weights, the measured confounders were balanced (standardized mean difference <0.1) between the new-users and nonusers (Supplementary Table S2). At the first follow-up visit after index, the association of gabapentin initiation with cognitive decline was statistically significant in CDRGLOB (OR [95% CI]: 1.55 [1.07, 2.25] and in CDR-SB (1.94 [1.22, 3.09]. At the second follow-up visit after index, the ORs were in the same direction but attenuated (CDRGLOB: 1.26 [0.84, 1.89]; CDR-SB: 1.57 [0.99, 2.47]) (Figure 3).

**TABLE 1:** Baseline characteristics in gabapentin new-users and non-users.

	Cognitive decline and functional status change analysis			Motor function change analysis		
	New-users (N = 480)	Nonusers (N = 4320)	*p* value	New-users (N = 459)	Nonusers (N = 4131)	*p* value
Year of enrollment N (%)						
2005	28 (5.8)	252 (5.8)		28 (6.1)	252 (6.1)	
2006	125 (26.0)	1125 (26.0)		119 (25.9)	1071 (25.9)	
2007	59 (12.3)	531 (12.3)		56 (12.2)	504 (12.2)	
2008	25 (5.2)	225 (5.2)		25 (5.5)	225 (5.5)	
2009	36 (7.5)	324 (7.5)		36 (7.8)	324 (7.8)	
2010	34 (7.1)	306 (7.5)		31 (6.8)	279 (6.8)	
2011	24 (5.0)	216 (5.0)		23 (5.0)	207 (5.0)	
2012	36 (7.5)	324 (7.5)		36 (7.8)	324 (7.8)	
2013	25 (5.2)	225 (5.2)		24 (5.2)	216 (5.2)	
2014	23 (4.8)	207 (4.8)		22 (4.8)	198 (4.8)	
2015	24 (5.0)	216 (5.0)		23 (5.0)	207 (5.0)	
2016	14 (2.9)	126 (2.9)		10 (2.2)	90 (2.2)	
2017	17 (3.5)	153 (3.5)		16 (3.5)	144 (3.5)	
2018	7 (1.5)	63 (1.5)		7 (1.5)	63 (1.5)	
2019	3 (0.6)	27 (0.6)		3 (0.7)	27 (0.7)	
Age N (%)			0.57			0.29
65–74 (years)	150 (31.3)	1453 (33.6)		141 (30.7)	1419 (34.4)	
75–84 (years)	225 (46.9)	1960 (45.4)		216 (47.1)	1861 (45.1)	
85+ (years)	105 (21.9)	907 (21.0)		102 (22.2)	851 (20.6)	
Male gender N (%)	165 (34.4)	1484 (34.4)	0.99	156 (34.0)	1434 (34.7)	0.76
Race N (%)			0.24			0.18
White	377 (78.5)	3518 (81.4)		358 (78.0)	3363 (81.4)	
Black	87 (18.1)	656 (15.2)		86 (18.7)	638 (15.4)	
Other	16 (3.3)	146 (3.4)		15 (3.3)	130 (3.2)	
Education N (%)			<0.0001			<0.0001
High school or less	115 (24.0)	645 (14.9)		110 (24.0)	653 (15.8)	
College degree	192 (40.0)	1847 (42.8)		184 (40.1)	1735 (42.0)	
Graduate degree	173 (36.0)	1821 (42.2)		165 (36.0)	1734 (42.0)	
Unknown	0 (0)	7 (0.2)		0 (0)	9 (0.2)	
Diabetes N (%)			<0.0001			<0.0001
Yes	106 (22.1)	512 (11.9)		103 (22.4)	485 (11.7)	
Unknown	165 (34.4)	1624 (37.6)		150 (32.7)	1500 (36.3)	
Hypertension N (%)			0.005			0.002
Yes	306 (63.8)	2441 (56.5)		296 (64.5)	2315 (56.0)	
Unknown	96 (20.0)	948 (21.9)		87 (19.0)	928 (22.5)	
Smoking history N (%)			0.06			0.024
Yes	244 (50.8)	1965 (45.5)		234 (51.0)	1849 (44.8)	
Unknown	2 (0.4)	37 (0.9)		2 (0.4)	42 (1.0)	
Depression N (%)	62 (12.9)	339 (7.9)	<0.0001	59 (12.9)	294 (7.1)	<0.0001
Parkinson’s disease N (%)	7 (1.5)	24 (0.6)		2 (0.4)	23 (0.6)	
Anxiety in the last month N (%)			0.02			
Yes	51 (10.6)	325 (7.5)		46 (10.0)	294 (7.1)	
Unknown	41 (8.5)	303 (7.0)		38 (8.3)	273 (6.6)	
Opioid N (%)	98 (20.4)	178 (4.1)	<0.0001	93 (20.3)	169 (4.1)	<0.0001
Antipsychotics N (%)			0.33			0.36
Yes	5 (1.0)	23 (0.5)		5 (1.1)	26 (0.6)	
Unknown	0 (0)	4 (0.1)		0 (0)	6 (0.2)	
Benzodiazepines N (%)	52 (10.8)	223 (5.2)	<0.0001	50 (10.9)	228 (5.5)	<0.0001
Anxiolytic, sedative, and hypnotics N (%)			<0.0001			<0.0001
Yes	104 (21.7)	478 (11.1)		101 (22.0)	468 (11.3)	
Unknown	0 (0)	4 (0.1)		0 (0)	6 (0.2)	
Antiseizures N (%)	27 (5.6)	96 (2.2)	<0.0001	27 (5.9)	96 (2.3)	<0.0001
Body Mass Index N (%)			<0.0001			0.0009
Normal	119 (24.8)	1467 (34.0)		115 (25.1)	1326 (32.1)	
Overweight	165 (34.4)	1413 (32.7)		160 (34.9)	1453 (35.2)	
Obese	140 (29.2)	921 (21.3)		132 (28.8)	881 (21.3)	
Underweight	3 (0.6)	47 (1.1)		3 (0.7)	58 (1.4)	
Unknown	53 (11.0)	472 (10.9)		49 (10.7)	413 (10.0)	
APOE e4 genotype N (%)			0.44			0.46
Yes	113 (23.5)	1128 (26.1)		108 (23.5)	1048 (25.4)	
Unknown	35 (7.3)	285 (6.6)		34 (7.4)	256 (6.2)	

### 3.2 Functional status decline

The same sample was used for assessing functional status change as measuring cognitive decline (Figure 1A). At the first visit after index, the association of gabapentin initiation with functional decline was not significant for either change in FAQ sum (OR [95% CI]: 1.50 [0.76, 2.96]) or FAQ mean (1.56 [0.93, 2.63]). At the second follow-up visit after index, the association of gabapentin initiation and decline in mean FAQ was statistically significant (1.78 [1.12, 2.83]) (Figure 3).

### 3.3 Motor function change

For measuring motor function change, participants who had gait disorders, falls, or slowness at the index visit were excluded from the sample. We identified 459 gabapentin new-users (78.7 [6.9]; 34.0%), and randomly selected 4,131 nonusers (78.2 [6.9]; 34.7%) (Figure 1B). Since only a small number of gabapentin initiators had motor dysfunction at index (n = 21), the mean (SD) number of annual visits between the first UDS visit and the index visit remained 4.8 (2.8). Gabapentin initiators reported more anxiety in the last month (new-users vs. non-user: 10.0% vs. 7.1%) and were more likely to have smoking history (i.e., at least 100 cigarettes) (51.0% vs. 44.8%), and to report using anxiolytic, sedative, and hypnotics (22.0% vs. 11.3%) and antiseizure medications (5.9% vs. 2.3%) than non-users (Table 1).

The association of gabapentin initiation with gait disorder was close to null at both the first (OR [95% CI]: 0.97 [0.44, 2.15]) and the second follow-up visit (1.10 [0.54, 2.24]) after index. For slowness, our results indicated increased odds of slowness in gabapentin initiators at the first (1.44 [0.70, 2.97]) and at the second follow-up visit after index (1.39 [0.66, 2.92]), but the results were not statistically significant. For falls, the association with gabapentin initiation was statistically significant (2.51 [1.19, 5.31]) at the second follow-up visit after index. At the first follow-up visit after index, the association of gabapentin initiation with falls was not statistically significant but was in the same direction (1.42 [0.61, 3.31]) (Figure 3).

## 4 Discussion

Using data from longitudinally followed cognitively normal older adult research volunteers, this study examined the association of gabapentin initiation with neurocognitive outcomes. Our results provide evidence that gabapentin was associated with increased odds of global cognitive decline, functional status decline, and motor function change (e.g., falls and slowness) in the 2 years following gabapentin initiation.

The results from this study are consistent with previous studies that found gabapentin use was associated with deleterious cognitive change. Shem *et al.* conducted a case series including ten patients with spinal cord injury. The results from this study showed that gabapentin therapy was associated with decline in memory, executive function, and attention after 1 week of gabapentin treatment (Shem et al., 2018). In a cross-over randomized controlled study, gabapentin use caused significantly worse attention/vigilance, ability to maintain wakefulness voluntarily, and cognitive processing and motor speed in healthy adults (Meador et al., 1999). A recent retrospective cohort study reported that gabapentin initiation in older adults after surgery was associated with increased risk of delirium and antipsychotic use (Park et al., 2022). However, several previous studies reported that gabapentin is not associated with cognitive functioning in patients with seizures (Leach et al., 1997; Dodrill et al., 1999). Although our study features a large, well-characterized cohort with multiple measures of neurocognitive function, our measure of gabapentin use was limited to self-report at annual visits. Therefore, studies with careful measurement of gabapentin use combined with careful measurements of neurocognitive outcomes are needed. Additionally, given the strong possibility of baseline differences in participants who and do not initiate gabapentin, observational studies must also make every effort to control confounding.

Antiseizure drugs are known to be associated with adverse cognitive effects *via* suppressing neuronal excitability or enhancing inhibitory neurotransmission (Martin et al., 1999; Ortinski and Meador, 2004; Loring et al., 2007; Eddy et al., 2011; Quon et al., 2020). However, gabapentin seems to be different from the traditional antiseizure drugs, and the exact mechanism(s) through which gabapentin exerts both its clinical and potential side effects is still unknown. One hypothesis includes binding to the alpha2-delta subunit of the voltage-dependent calcium channel (Rose and Kam, 2002). Considering that gabapentin may block calcium channels in the brain, it is possible that it would have a neuroprotective effect, but this is controverted by the results of this study. Therefore, further experimental studies are needed to examine the mediators of gabapentin use and neurocognitive changes.

In addition to the neurocognitive outcomes under study, we also found that the gabapentin initiators had higher prevalence of opioid use, as well as antidepressants, antipsychotics, benzodiazepines, and anxiolytics, sedatives, and hypnotics compared to nonusers. This result is consistent to our previous study that examined the concurrent use of gabapentin with CNS-depressant medications (Oh et al., 2022). As the FDA and the Beers 2019 criteria warn about using gabapentin concurrently with some medications due to risk of respiratory depression (U.S. Food and Drug Administration, 2019; By the 2019 American Geriatrics Society Beers Criteria® Update Expert Panel, 2019), further studies are needed to examine the risk of concurrently using gabapentin with other CNS depressants in older adults.

This study has several strengths and limitations. First, the NACC UDS dataset has rich data, including participant medical history and neurocognitive evaluations. Using this resource, we had greater sample size compared to the previous studies and were able to measure the association of gabapentin initiation and neurocognition with various clinically relevant outcomes. To mitigate confounding and selection bias, we employed a new-user design and inverse probability weighting. Also, non-users were randomly selected matching on the year of first enrollment and the number of visits from enrollment to gabapentin initiation to new-users to minimize bias (e.g., secular trends, survival bias, and attrition bias). However, gabapentin initiators were identified by reported medication used within 2 weeks of their UDS visit, so participants could be misclassified as non-users if they used gabapentin only between visits. There is less probability of misclassifying a non-user as a user in this setting. Although we used causal diagrams to select the set of essential confounders, we note that residual confounding (Suttorp et al., 2015), such as unmeasured (e.g., drug indication), partially measured (e.g., seizure and arthritis), and unknown confounders, remains. In the minimum set of confounders identified by DAG, seizure and arthritis were included for measuring cognitive and functional status decline and for measuring motor function change, respectively. However, seizure and arthritis were only partially measured in our study sample (seizure: 57.8%; arthritis: 40.6%) due to changes in the data collection protocol. Thus, these variables were not included in our model. Additionally, participants in the NACC dataset tend to be highly educated and white race, which limits generalizability.

In conclusion, this study showed that among older adults with normal cognition, initiating gabapentin was significantly associated with clinically meaningful decline in cognitive and functional status and increased falls. Further studies are needed to examine the risk and benefit of prescribing gabapentin in older adults.

## Data Availability

The datasets presented in this article are not readily available because the data was obtained from the National Alzheimer’s Coordinating Center. Requests to access the datasets should be directed to: https://naccdata.org/requesting-data/submit-data-request.
