# Supplementary material for: The association of gabapentin initiation and neurocognitive changes in older adults with normal cognition
Source: Front Pharmacol. 2022 Nov 25;13:910719. doi: 10.3389/fphar.2022.910719 (PMC9732650; doi:10.3389/fphar.2022.910719)
Supplement: Supplementary file 1 [file DataSheet1.DOCX]

**Table S1. Description of variables used in the study**

| Variables | Form | Description |
| --- | --- | --- |
| Age | A1 subject demographics | Subject’s age at visit |
| Sex | A1 subject demographics | Subject’s sex |
| Education | A1 subject demographics | Years of education (0-36)  12=high school or GRE  16=Bachelor’s degree  18=Master’s degree  20=Doctorate |
| Race | A1 subject demographics | White  Black or African American  Other (American Indian, Alaska Native, Native Hawaiian, Pacific Islander, Asian, or Other)  Unknown |
| Smoking history | A5 subject health history | Smoked more than 100 cigarettes in life |
| Body mass index | B1 Physical | Body mass index  0 < BMI <18.5: Under  18.5 ≤ BMI <25: Normal  25≤BMI<30: Overweight  30≤BMI: Obesity |
| Depression | D1 clinician diagnosis | Presumptive etiologic diagnosis |
| Diabetes | A5 subject health history | Diabetes ever vs. never |
| Hypertension | A5 subject health history | Hypertension ever vs. never |
| Parkinson’s disease | D1 clinician diagnosis | Parkinson’s disease present |
| Anxiety | B5 Neuropsychiatric Inventory Questionnaire | Anxiety in the last month |
| Opioids | A4 subject medications | 'acetaminophen-codeine' 'acetaminophen-hydrocodone' 'acetaminophen-oxycodone' 'acetaminophen-tramadol' 'acetaminophen/butalbital/caffeine/codeine'  'acetaminophen/caffeine/dihydrocodeine' 'asa/caffeine/propoxyphene' 'aspirin/butalbital/caffeine/codeine'  'aspirin/caffeine/dihydrocodeine' 'aspirin/carisoprodol/codeine' 'aspirin-hydrocodone' 'aspirin-oxycodone'  'bupivacaine-fentanyl' 'bupivacaine-hydromorphone' 'buprenorphine'  'codeine'  'dihydrocodeine' 'droperidol-fentanyl' 'fentanyl' 'fentanyl topical' 'fentanyl-ropivacaine'  'hydrocodone' 'hydrocodone-ibuprofen'  'hydromorphone' 'meperidine' 'methadone' 'morphine' 'opium' 'oxycodone' 'oxymorphone' 'propoxyphene' 'tramadol' |
| Antiseizure | A4 subject medications | 'phenytoin' 'phenobarbital' 'carbamazepine' 'oxcarbazepine' 'pregabalin' 'lacosamide'  'valproic acid' 'lamotrigine' 'topiramate' 'zonisamide' 'levetiracetam' 'clonazepam' 'rufinamide' |
| Anxiolytic, sedative, and hypnotic | A4 subject medications | Reported current use of an anxiolytic, sedative, or hypnotic agent |
| Antipsychotic | A4 subject medications | Reported current use of antipsychotic agent |
| Benzodiazpine | A4 subject medications | 'alprazolam' 'chlordiazepoxide' 'chlordiazepoxide-clidinium' 'chlordiazepoxide-methscopolamine' 'clobazam' 'clonazepam' 'clorazepate' 'diazepam' 'estazolam' 'flurazepam'  'halazepam' 'lorazepam' 'oxazepam' 'prazepam' 'quazepam' 'temazepam' 'triazolam |
| APOE e4 | Genetic data | Having one or two copies of e4 allele from the nACC derived variable |


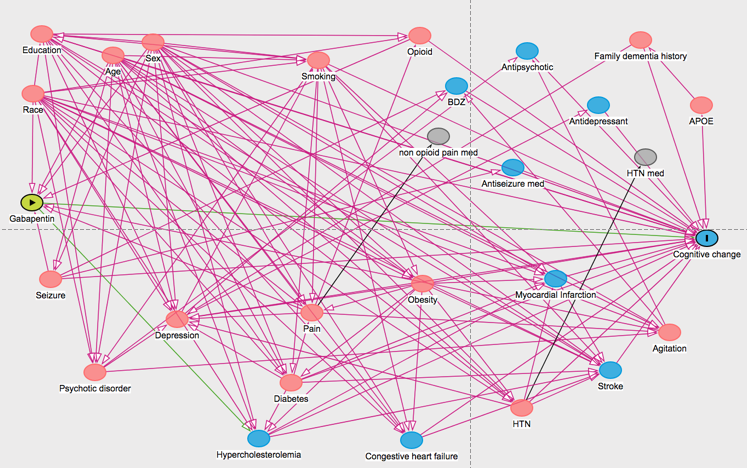


**Figure 1S. Directed acyclic graphs for Cohort A**

**
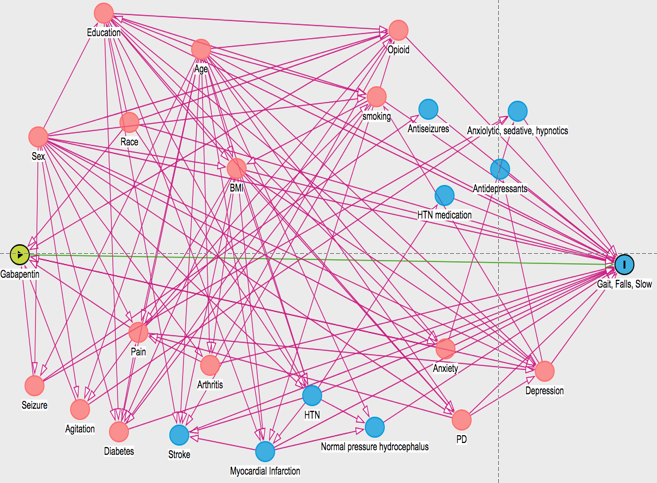
**

**Figure 2S. Directed acyclic graphs for Cohort B**

**Table S2. Standardized mean differences between new-users and non-users in unweighted and weighted sample for measuring cognitive decline and functional status change**

|  | Unweighted | Weighted |
| --- | --- | --- |
| Age | 0.052 | 0.016 |
| Sex | 0 | 0.002 |
| Race | 0 | 0.002 |
| Education | 0 | 0.002 |
| Depression | 0.167 | 0.047 |
| Diabetes | 0.167 | 0.047 |
| Hypertension | 0.167 | 0.047 |
| Smoking history | 0.167 | 0.047 |
| Opioid | 0.513 | 0.018 |
| Antipsychotics | 0.167 | 0.047 |
| Benzodiazepine | 0.21 | 0.056 |
| Body Mass Index | 0.167 | 0.047 |
| APOE e4 genotype | 0.167 | 0.047 |
